# Supplementary material for: Protective effect of Enterococcus faecium against ethanol-induced gastric injury via extracellular vesicles
Source: Microbiol Spectr. 2024 Mar 15;12(4):e03894-23. doi: 10.1128/spectrum.03894-23 (PMC10986489; doi:10.1128/spectrum.03894-23)
Supplement: Supplemental Tables — Microbial species identification results and sequences of primers for real-time PCR. [file spectrum.03894-23-s0001.docx]

Supplementary Material

Supplementary Tables

**Table S1** Species identification result

| **Description** | **Scientific Name** | **Query Cover** | **Accession** |
| --- | --- | --- | --- |
| Enterococcus faecium strain DT1-1 chromosome, complete genome | Enterococcus faecium | 100% | CP050255.1 |
| Enterococcus faecium strain DMEA02 chromosome, complete genome | Enterococcus faecium | 100% | CP043484.1 |
| Enterococcus faecium strain FA3 chromosome, complete genome | Enterococcus faecium | 100% | CP042834.1 |
| Enterococcus sp. DA9 chromosome, complete genome | Enterococcus sp. DA9 | 100% | CP042839.1 |
| Enterococcus faecium strain HB-1 chromosome, complete genome | Enterococcus faecium | 100% | CP040878.1 |
| Enterococcus faecium strain Gr17 chromosome, complete genome | Enterococcus faecium | 100% | CP033376.1 |
| Enterococcus faecium strain LMEM 41 16S ribosomal RNA gene, partial sequence | Enterococcus faecium | 100% | MK418596.1 |
| Enterococcus faecium strain LMEm 40 16S ribosomal RNA gene, partial sequence | Enterococcus faecium | 100% | MK418593.1 |
| Enterococcus faecium strain LMEM 34 16S ribosomal RNA gene, partial sequence | Enterococcus faecium | 100% | MK418583.1 |
| Enterococcus faecium strain LMEM3 16S ribosomal RNA gene, partial sequence | Enterococcus faecium | 100% | MK418578.1 |

>S082-2 1493bp

TCAGGACGAACGCTGGCGGCGTGCCTAATACATGCAAGTCGTACGCTTCTTTTTCCACCGGAGCTTGCTCCACCGGAAAAAGAGGAGTGGCGAACGGGTGAGTAACACGTGGGTAACCTGCCCATCAGAAGGGGATAACACTTGGAAACAGGTGCTAATACCGTATAACAATCGAAACCGCATGGTTTTGATTTGAAAGGCGCTTTCGGGTGTCGCTGATGGATGGACCCGCGGTGCATTAGCTAGTTGGTGAGGTAACGGCTCACCAAGGCCACGATGCATAGCCGACCTGAGAGGGTGATCGGCCACATTGGGACTGAGACACGGCCCAAACTCCTACGGGAGGCAGCAGTAGGGAATCTTCGGCAATGGACGAAAGTCTGACCGAGCAACGCCGCGTGAGTGAAGAAGGTTTCGGATCGTAAAACTCTGTTGTTAGAGAAGAACAAGGATGAGAGTAACTGTTCATCCCTTGACGGTATCTAACCAGAAAGCCACGGCTAACTACGTGCCAGCAGCCGCGGTAATACGTAGGTGGCAAGCGTTGTCCGGATTTATTGGGCGTAAAGCGAGCGCAGGCGGTTTCTTAAGTCTGATGTGAAAGCCCCCGGCTCAACCGGGGAGGGTCATTGGAAACTGGGAGACTTGAGTGCAGAAGAGGAGAGTGGAATTCCATGTGTAGCGGTGAAATGCGTAGATATATGGAGGAACACCAGTGGCGAAGGCGGCTCTCTGGTCTGTAACTGACGCTGAGGCTCGAAAGCGTGGGGAGCAAACAGGATTAGATACCCTGGTAGTCCACGCCGTAAACGATGAGTGCTAAGTGTTGGAGGGTTTCCGCCCTTCAGTGCTGCAGCTAACGCATTAAGCACTCCGCCTGGGGAGTACGACCGCAAGGTTGAAACTCAAAGGAATTGACGGGGGCCCGCACAAGCGGTGGAGCATGTGGTTTAATTCGAAGCAACGCGAAGAACCTTACCAGGTCTTGACATCCTTTGACCACTCTAGAGATAGAGCTTCCCCTTCGGGGGCAAAGTGACAGGTGGTGCATGGTTGTCGTCAGCTCGTGTCGTGAGATGTTGGGTTAAGTCCCGCAACGAGCGCAACCCTTATTGTTAGTTGCCATCATTCAGTTGGGCACTCTAGCAAGACTGCCGGTGACAAACCGGAGGAAGGTGGGGATGACGTCAAATCATCATGCCCCTTATGACCTGGGCTACACACGTGCTACAATGGGAAGTACAACGAGTTGCGAAGTCGCGAGGCTAAGCTAATCTCTTAAAGCTTCTCTCAGTTCGGATTGCAGGCTGCAACTCGCCTGCATGAAGCCGGAATCGCTAGTAATCGCGGATCAGCACGCCGCGGTGAATACGTTCCCGGGCCTTGTACACACCGCCCGTCACACCACGAGAGTTTGTAACACCCGAAGTCGGTGAGGTAACCTTTTTGGAGCCAGCCGCCTAAGGTGGGATAGATGATTGGGGTGAAGTCGT

**Table S2** Sequences of primers for real-time PCR

| Gene Name | GenBank accession No. | Sequences of primers (5′–3′) | Annealing temperature, ℃ | PCR product size, bp |
| --- | --- | --- | --- | --- |
| *β-actin* | NM_031144.3 | F: CCGTAAAGACCTCTATGCCAACA | 58.7 | 230 |
|  |  | R: CGGACTCATCGTACTCCTGCTT |  |  |
| *ET-1* | NM_012548.2 | F: AGCAACAGCATCAAGACCTCC | 61 | 123 |
|  |  | R: TCATGGCTTCCAAAACACCAC |  |  |
| *Muc1* | NM_001398538.1 | F: CCACACTCACGGACGCTATGT | 61 | 151 |
|  |  | R: GTGGGTAGGGTGACTTGCTCC |  |  |
| *Muc6* | XM_039098009.1 | F: GGAACCCCATAAGTACGCTAC | 58.7 | 115 |
|  |  | R: GTTGCAGATATGTGCGTATCT |  |  |
| *Nfkb1* | NM_001415012.1 | F: GCATTCTGACCTTGCCTAT | 61 | 183 |
|  |  | R: TCCAGTCTCCGAGTGAAGC |  |  |
| *IL-1b* | NM_031512.2 | F: TGAACTCAACTGTGAAATAGC | 58.7 | 277 |
|  |  | R: AAAGATGAAGGAAAAGAAGGT |  |  |
| *IL-6* | NM_012589.2 | F: TTTGCCTATTGAAAATCTGCTC | 61 | 231 |
|  |  | R: GCCACTCCTTCTGTGACTCTAA |  |  |
| *IL-10* | XM_006249712.4 | F: CCATAGGGGACATTTTATAGTATTT | 61 | 103 |
|  |  | R: GCCTTAGGATCGAAGTTTCAG |  |  |
